# Robot-Assisted Microsurgery Has a Steeper Learning Curve in Microsurgical Novices

**DOI:** 10.3390/life15050763

**Published:** 2025-05-09

**Authors:** Felix Struebing, Jonathan Weigel, Emre Gazyakan, Laura Cosima Siegwart, Charlotte Holup, Ulrich Kneser, Arne Hendrik Boecker

**Affiliations:** BG Trauma Center Ludwigshafen, Department for Plastic, Hand and Reconstructive Surgery, Department of Plastic Surgery for the University of Heidelberg, Ludwig Guttmann-Strasse 13, 67071 Ludwigshafen, Germany; felix.struebing@bgu-ludwigshafen.de (F.S.);

**Keywords:** robot-assisted microsurgery, learning curve, surgical education, robotic assistance, microsurgery

## Abstract

Introduction: Mastering microsurgery requires advanced fine motor skills, hand–eye coordination, and precision, making it challenging for novices. Robot-assisted microsurgery offers benefits, such as eliminating physiological tremors and enhancing precision through motion scaling, which may potentially make learning microsurgical skills easier. Materials and Methods: Sixteen medical students without prior microsurgical experience performed 160 anastomoses in a synthetic model. The students were randomly assigned into two cohorts, one starting with the conventional technique (HR group) and one with robotic assistance (RH group) using the Symani surgical system. Results: Both cohorts showed a reduction in procedural time and improvement in SAMS scores over successive attempts, with robotic anastomoses demonstrating a 48.2% decrease in time and a 54.6% increase in SAMS scores. The decreases were significantly larger than the RH group (*p* < 0.05). The quality of the final anastomoses was comparable in both groups (*p* > 0.05). Discussion: This study demonstrated a steep preclinical learning curve for robot-assisted microsurgery (RAMS) among novices in a synthetic, preclinical model. No significant differences in SAMS scores between robotic and manual techniques after ten anastomoses. Robot-assisted microsurgery required more time per anastomosis, but the results suggest that experience with RAMS may aid in manual skill acquisition. The study indicates that further exploration into the sequencing of robotic and manual training could be valuable, especially in designing structured microsurgical curricula.

## 1. Introduction

Except for improvements in optical magnification systems, there have only been relatively few advancements in the technical aspects of microsurgery. The learning process involves the acquisition of fine motor skills, hand–eye coordination, and the ability to work under a microscope [1,2]. These skills are challenging and time-consuming to master, especially for novices [3]. This is especially true for the field of supermicrosurgery, which deals with the anastomosis of vessels with a diameter of less than 1 mm [4]. These extremely small structures require an even more delicate technique with atraumatic tissue handling, high precision, and a steady hand.

Recently, robot-assisted microsurgery (RAMS) has been introduced to the field of plastic surgery [5,6]. Benefits of robot-assisted microsurgery include, besides ergonomic improvements, the elimination of the physiologic tremor and motion scaling, enabling a very high degree of precision. The preclinical learning curve using the Symani surgical system (Medical Microinstruments Inc., Jacksonville, FL, USA) has been previously described by several authors. It has been shown to be quite steep in both the hands of experienced microsurgeons and microsurgical novices [7,8,9,10]. In previous experimental studies, novices performed microsurgical anastomoses using only the Symani system or alternating between the conventional technique and robotic assistance. Wessel et al. did not find significant differences in anastomosis time or quality after a series of ten anastomoses performed by microsurgical novices [9]. Cho and colleagues demonstrated that the anastomosis quality became statistically equivalent after a total of eight anastomoses, but that RAMS required more time than conventional technique [10].

So far, two other robotic systems have been utilized for microsurgical applications: the DaVinci (Intuitive Surgical Inc., Sunnyvale, CA, USA) and the Microsure MUSA systems (Microsure, Eindhoven, The Netherlands). DaVinci is a robotics platform that is mostly utilized in general surgery, urology, and obstetrics, but may be used for microsurgery as well [11,12,13]. MUSA is a robotics platform specifically designed for microsurgery. It has been used in a series of lymphovenous anastomoses in clinical practice [14,15,16].

We hypothesize that robotic assistance may aid in reducing the learning curve for future microsurgeons and possibly lead to more widespread utilization of supermicrosurgical techniques. To date, no studies have evaluated whether the timing of introducing robotic assistance into the microsurgical curriculum—either immediately or following an introductory period to conventional microsurgery—affects outcomes.

Therefore, we designed a prospective, experimental, preclinical study analyzing whether robot-assisted microsurgery is as easily learned as the conventional technique by microsurgical novices. For that purpose, two randomized groups of medical students who had no prior experience in microsurgery were established. One group started with the conventional hand-sewn technique, while the other started with robotic assistance. After a series of five anastomoses, the techniques were switched, and an additional five anastomoses were performed.

## 2. Material and Methods

### 2.1. Study Design

An a priori power analysis was performed (the primary endpoint was defined as the SAMS score of the final anastomosis and the delta as 0.5 ± 0.3), resulting in a group size of 8. All 16 participants were medical students from the medical faculty of Mannheim (University Heidelberg). Eligibility was determined through a screening against predefined inclusion/exclusion criteria prior to enrollment. Participants had to be medical students with sufficient language proficiency. Individuals with previous microsurgical experience were ruled out. Qualified candidates were randomized into two cohorts using computer-generated randomization (random.org accessed first on 2 February 2024) with a 1:1 allocation ratio. One cohort started their microsurgical training with the conventional technique (hand–robot group; HR group), while the other cohort started with robot-assisted microsurgery (robot–hand group; RH group).

**Figure 1:**
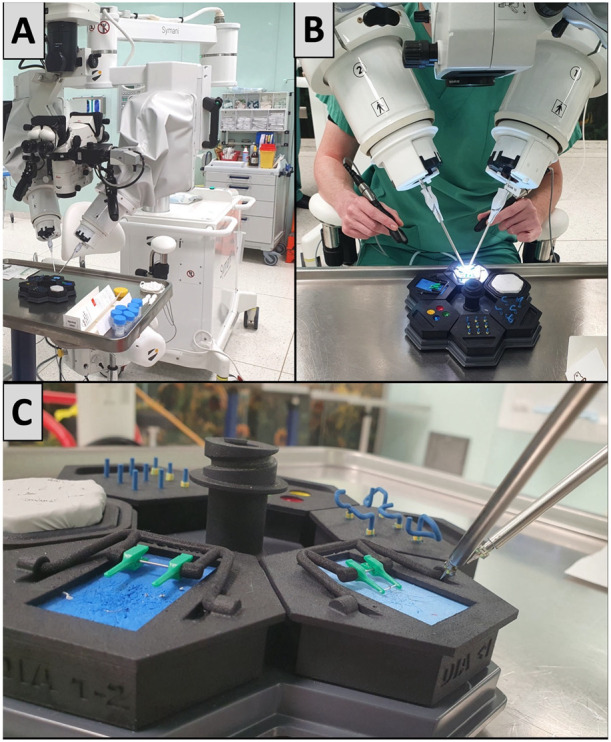
(**A**) The experimental setup using the Symani surgical system and a conventional operating microscope. (**B**) A robot-assisted anastomosis is performed; the robotic instruments are controlled by the participant holding the two forceps-like controllers. (**C**) Close-up of the robotic instruments and the vascular clamps holding an artificial vessel after completion of an anastomosis.

Prior to the first session, all participants completed a questionnaire capturing demographic and anamnestic data (previous surgical and microsurgical experience, handedness, video game exposure) and received an overview of basic microsurgical principles. For the HR group, this introduction comprised an explanation of standard microsurgical instruments and their handling, a video-guided demonstration of the microvascular anastomosis technique, and a standardized hands-on session in which each participant tied ten microsurgical knots on a silicone-glove model. The RH group received an explanation of the Symani Surgical System and its robotic instruments, followed by the same instructional video on basic microsurgical anastomosis. They subsequently practiced basic robotic instrument handling and dexterity in a series of structured exercises (see Figure 1C) before advancing to microsuturing. Before switching techniques, the relevant instruments were introduced and the corresponding hands-on practice was completed without repeating the video introduction.

Five hand-sewn and robotic anastomoses were completed by each group over the course of approximately five appointments. The HR group proceeded with the robotic anastomoses and the RH group with the manual anastomoses after completing five anastomoses with the other microsurgical technique. Figure 1 depicts the experimental setup for the robotic anastomoses. Manual anastomoses were performed in the same setting, except for using conventional micro-instruments instead of the Symani system.

All participants were instructed to use the open-book technique for microsurgical anastomoses. In this technique, the posterior wall is sutured first using interrupted stitches with the lumen fully exposed, reducing the risk for back-wall stitches. The anterior wall is sutured using interrupted stitches. No traction sutures were used. Participants did not receive any physical assistance during microsurgical anastomoses (e.g., thread cutting or suture handling). However, verbal guidance by an experienced instructor was provided, and participants of both groups were allowed to ask questions whenever uncertainties arose.

Synthetic vessels measuring 2.0 mm in diameter (Wetlab Incorporated, Shiga, Japan) and Ethicon Ethilon 8-0 monofilament nylon sutures (Johnson & Johnson MedTech, New Brunswick, NJ, USA) were utilized for all anastomoses. A conventional operating microscope was used for all sessions (Mitaka MM51, Mitaka Kohki Ltd., Tokyo, Japan, or Leica F50, Leica Microsystems GmbH, Wetzlar, Germany). The robot-assisted anastomoses were performed using the Symani surgical system (Medical Microinstruments, Inc., Wilmington, DE, USA) with the microscope positioned between the robot arms. All anastomoses were recorded using the integrated video camera of the surgical microscope and later assessed by a single assessor using the SAMS score for microsurgical anastomoses. The assessor was aware of each participant’s group allocation and attempt number.

### 2.2. SAMS Score

The SAMS score, initially described by Chan et al. is a structured, objective assessment method of microsurgical skills and proficiency [17].

It features three assessment components: a global rating scale, an error checklist, and an overall summative rating. The global rating scale consists of four categories (dexterity, visuospatial ability, operative flow, and judgment), each subdivided into three specific microsurgical skills rated on a five-point scale. Not all items were applicable in our study setting: specifically, the items dissection, irrigation, patency test, and bleeding control were omitted because synthetic vessels without perfusion were used in the study, requiring no prior dissection. After each completed anastomosis, a summative rating is given on a five-point scale (1 = bad; 5 = excellent). The third assessment component of the SAMS score is an error checklist with 25 predefined technical errors categorized into different microsurgical skill domains. From this list, we excluded ten checklist errors that were not applicable to our study. Specifically, the entire ‘planning’ domain and six additional items—vessel–clamp reapplication, insufficient vessel preparation, inappropriate magnification, vessel desiccation, fluid pooling, and the ‘crushing’ patency test—were omitted. These omissions reflect our experimental design, in which focus, field of view, and vessels were prepared in advance, and participants were not responsible for intraoperative irrigation.

### 2.3. Statistical Analysis

The Welch t-test was used for the analysis of normally distributed data and the Mann–Whitney U test for not normally distributed data. Categorical variables were analyzed using chi-square testing. Data normality was assessed using the Shapiro–Wilk test. Correlation analysis was carried out using the Spearman Correlation Test. Continuous variables are presented with mean and standard deviation (SD) or median and interquartile range (IQR). Categorical variables are displayed with frequencies and percentages. Statistical significance was set at a *p*-value of 0.05 (two-tailed). All analyses were conducted in GraphPad Prism v9.0.2 (GraphPad Software, San Diego, CA, USA).

## 3. Results

### 3.1. Study Cohort

Sixteen medical students participated in the study, with eight randomly assigned to each cohort. The study cohort included nine male participants (56.3%) and seven female participants (43.7%). The mean age was 24.4 ± 3.2 years. Participants were in different stages of their undergraduate medical education, spanning first-year to last-year medical students. Handedness distribution revealed 14 right-handed individuals (87.5%) and two ambidextrous participants (12.5%); no left-handed participants were enrolled. Demographic characteristics are detailed in Table 1.

### 3.2. Anastomosis Characteristics

A total of 160 anastomoses were performed. There was an equal distribution of robot-assisted and conventionally hand-sewn anastomoses (n = 80 each). Synthetic vessels (Wetlab Incorporated, Shiga, Japan) measuring 2.0 mm in diameter were utilized for all anastomoses.

The median stitch count per anastomosis was 6 (IQR = 1), with no statistically significant difference observed between robotic and manual techniques (*p* = 0.37, Cohen’s d = 0.15, 95% CI [0.0, 0.0]). Robotic anastomoses required significantly more time per stitch and total anastomosis time than hand-sewn counterparts (time per stitch: *p* < 0.0001, d = 1.19, 95% CI [1.5, 2.5]; total time: *p* < 0.0001, d = 1.15, 95% CI [9.0, 16.0]). On average, the participants needed 57 percent more time to perform a robotic anastomosis (difference between means ± SEM: 14.7 ± 2.0 min).

**Figure 2:**
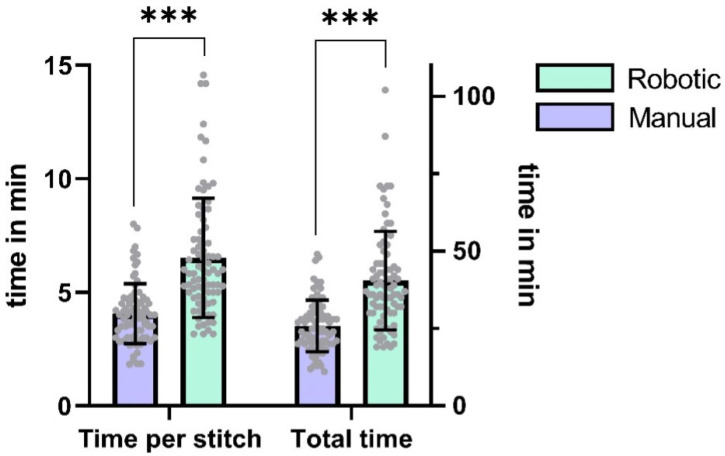
Comparison of mean anastomosis time and time per stitch (in min) for the two cohorts and the different microsurgical techniques (*p* < 0.0001 for both). Error bars represent standard deviation. *** *p* < 0.005.

Table 2 contains detailed information on the anastomosis characteristics. Figure 2 depicts the comparison of the different microsurgical techniques.

**Figure 3:**
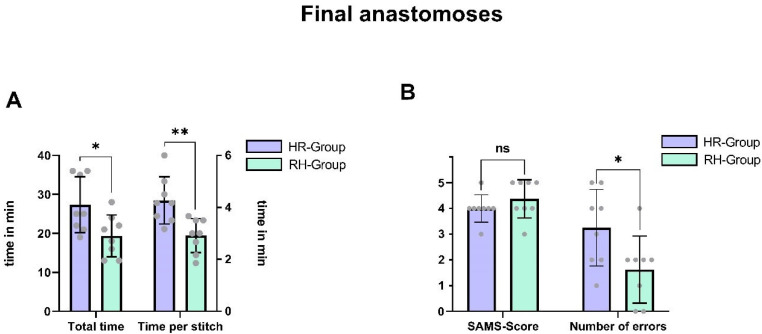
(**A**) Comparison of mean anastomosis time (*p* = 0.025) and time per stitch (in min) for the last anastomosis performed by each cohort. Error bars represent standard deviation. (**B**) Comparison of mean SAMS score (*p* = 0.15) and number of errors (*p* = 0.04) assessed by the SAMS checklist for the last anastomosis performed by each cohort. Error bars represent standard deviation. * *p* < 0.05, ** *p* < 0.01, ns non-significant.

When comparing the final anastomoses between cohorts, the differences were more nuanced. The last anastomosis performed robotically by the HR group required significantly more time than the last anastomosis performed manually by the robotic-first cohort (mean difference ± SEM: 8.0 ± 3.2 min, *p* = 0.025, d = 1.27, 95% CI [1.2, 14.8]). However, the SAMS score was only slightly higher in the RH group, and this difference did not reach statistical significance (mean difference ± SEM: 0.38 ± 0.3 points, *p* = 0.25, d = 0.58, 95% CI [−1.00, 0.00]). Figure 3 depicts the comparison of the final anastomoses per cohort.

**Figure 4:**
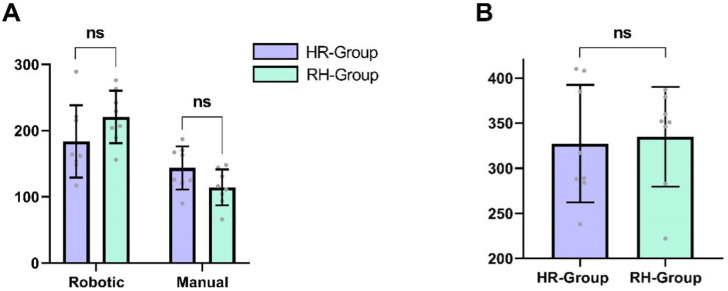
(**A**) Comparison of mean total time (in min) needed to complete five robotic/manual anastomoses. Error bars indicate standard deviation. (**B**) Comparison of mean total time (in min) needed to complete all ten anastomoses. Error bars indicate standard deviation. ns non-significant.

No significant difference was observed between cohorts regarding the total time required to complete all ten anastomoses (HR group: 327.4 ± 65.1 min, RH group: 335.0 ± 55.3 min, *p* = 0.99, d = 0.13, 95% CI [−72.0, 58.0]). When analyzing robotic and manual anastomoses separately, participants in the RH group required an average of 37.0 min longer to complete the five robotic anastomoses compared to the HR group (mean difference ± SEM: 37.0 ± 23.9 min, *p* = 0.15, d = 0.78, 95% CI [−88.7, 14.7]). During the final anastomosis, participants in the RH group made significantly fewer errors (*p* = 0.04, d = 1.28, 95% CI [0.12, 3.13]). Conversely, participants in the HR group took on average 29.4 min longer to complete the five manual anastomoses compared to the RH group (mean difference ± SEM: 29.4 ± 15.1 min, *p* = 0.07, d = 0.97, 95% CI [−3.0, 61.8]). However, neither of these differences reached statistical significance. Figure 4 depicts the total time taken for all anastomoses per cohort.

**Figure 5:**
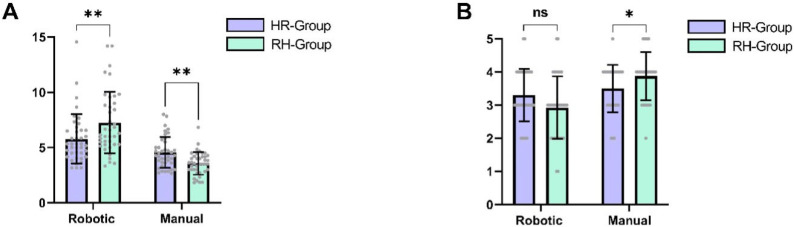
(**A**) Comparison of mean time per stitch (in min) between the two groups for the different microsurgical techniques. Error bars indicate standard deviation. (**B**) Comparison of mean SAMS score between the two groups for the different microsurgical techniques (manual: *p* = 0.03; robotic: *p* = 0.06). Error bars indicate standard deviation. * *p* < 0.05, ** *p* < 0.01, ns non-significant.

A similar pattern was observed for the SAMS scores. The RH group achieved significantly higher SAMS scores in manual anastomoses compared to the HR group (mean difference ± SEM: 0.38 ± 0.11 points, *p* = 0.03, d = 0.52, 95% CI [−1.0, 0.0]). Conversely, the HR group achieved slightly higher SAMS scores in robotic anastomoses, although this difference did not reach statistical significance (mean difference ± SEM: 0.38 ± 0.13 points, *p* = 0.06, d = 0.43, 95% CI [0.0, 1.0]). Figure 5 shows the time required per suture and SAMS scores for both techniques, stratified by group.

### 3.3. Learning Curve

**Figure 6:**
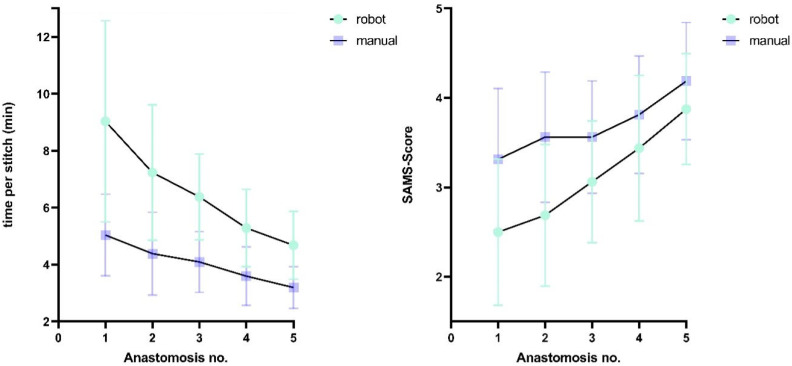
Time per stitch and SAMS score across successive anastomoses using robotic (green) and manual (purple) techniques. Error bars indicate standard deviation.

A progressive reduction in procedural time was observed across attempts: robotic stitches required 48.2% more time (mean difference: 4.4 min) during initial anastomoses compared to final attempts, while the conventional technique showed a 36.4% decrease (mean difference: 1.8 min). Similarly, SAMS scores improved by 54.6% (1.4 points) for final robotic anastomoses and 25.8% for manual anastomoses relative to first attempts. Figure 6 depicts the learning curves of the different techniques.

Cohort-specific analyses revealed differing time reductions and SAMS score changes for the different microsurgical techniques: the RH group showed significantly greater improvement for the time used per stitch in robotic anastomoses (*p* = 0.04, d = 0.91, 95% CI [−5.9, −0.3]), while the HR group exhibited significantly larger manual anastomosis gains (*p* = 0.04, d = 1.1, 95% CI [0.1, 2.1]). Although SAMS score trends paralleled these temporal patterns with a greater increase in the HR group for manual anastomoses and in the RH group for robotic anastomoses, inter-cohort differences did not reach statistical significance (robotic: *p* = 0.22, d = 0.86, 95% CI [−1.0, 0.0]; manual: *p* = 0.369, d = 0.63, 95% CI [0.0, 1.0]).

Notably, the RH group outperformed the HR group in initial manual anastomoses, requiring fewer minutes per stitch (difference between means: 1.6 min, *p* = 0.03, d = 1.31, 95% CI [0.2, 2.9]) and achieving a higher SAMS score (difference between means: 0.88, *p* = 0.03, d = 1.3, 95% CI [−1.6, −0.1]).

**Figure 7:**
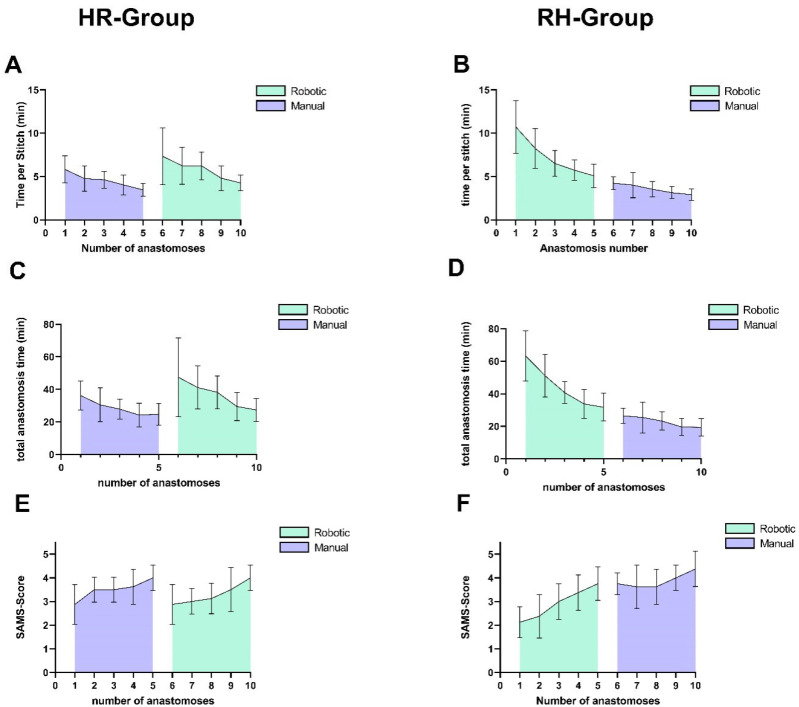
(**A**,**B**) Mean time per stitch (min) across successive anastomoses using robotic (green) and manual (purple) techniques for the different cohorts. Error bars indicate standard deviation. (**C**,**D**) Mean total anastomosis time (min) across successive anastomoses using robotic (green) and manual (purple) techniques for the different cohorts. Error bars indicate standard deviation. (**E**,**F**) Mean SAMS score across successive anastomoses using robotic (green) and manual (purple) techniques for the different cohorts. Error bars indicate standard deviation.

The first robot-assisted anastomoses performed by the HR group did not show significantly shorter times or better scores; however, there was a trend towards faster stitching (difference between means: 3.39 min, *p* = 0.08, d = 1.07, 95% CI [−7.6, 0.4]) and scoring higher on the SAMS scale (difference between means: 0.75, *p* = 0.13, Cohen’s d = 1.01, 95% CI [0.0, 2.0]), without reaching statistical significance (*p* > 0.05). Table 3 and Table 4 contain more detailed information on the anastomoses. Figure 7 depicts the learning curve of both cohorts.

### 3.4. Microsurgical Performance Factors

**Figure 8:**
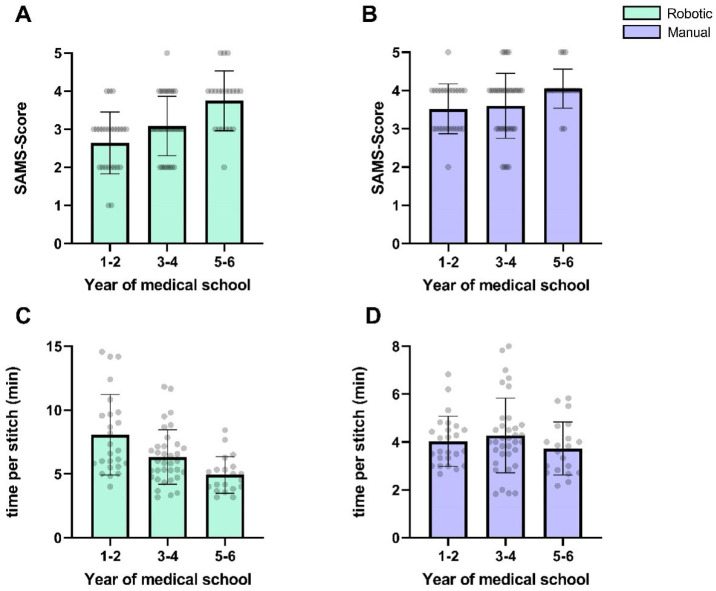
Mean time per stitch (**A**,**B**) and SAMS score (**C**,**D**) for participants grouped by year of medical school curriculum. Error bars indicate standard deviation.

Participants’ progression within their medical school curriculum showed a non-significant negative correlation with the time per stitch (r = −1.0, *p* = 0.33) and a positive correlation with SAMS scores (r = 1.0, *p* = 0.33). For manual anastomoses, the correlation with the SAMS score was less pronounced (r = 1.0; *p* = 0.33), while no meaningful correlation was observed for the time per stitch (r = −0.5; *p* > 0.99). Figure 8 depicts the microsurgical performance stratified by the participants’ medical school progression.

No significant correlation was identified between participants’ exposure to video games and the time per stitch required for either robotic or manual anastomoses (r = 0.3; *p* = 0.68). For the SAMS score, the correlation was also non-significant (r = −0.1; *p* = 0.95).

## 4. Discussion

In our experimental, preclinical study, we demonstrated a steep learning curve for robot-assisted microsurgical skills among microsurgical novices in a standardized synthetic model using the Symani system and 2 mm vessels with 8-0 sutures. The aim of the study was to evaluate the impact of the sequence of introduction to either conventional or robot-assisted microsurgery on overall performance in microsurgical novices. After five anastomoses, no statistically significant difference was observed in the quality of these anastomoses, as assessed by the SAMS score, regardless of whether students began with robotic or conventional microsurgery.

Students who began with the robotic system required more time for their first anastomoses but progressed rapidly thereafter. Their learning curve continued with minimal disruption upon switching to the conventional technique (see Figure 7B,D,F). In contrast, students who started with the conventional technique were initially faster but almost re-established their learning curve when transitioning to the robotic system (see Figure 7A,C,E). The initial manual anastomoses significantly outperformed the initial manual anastomoses in the HR group, while the first robot-assisted anastomoses of the HR group did not outperform the other cohort. This asymmetry in skill transfer supports the idea that starting with a more complex interface (such as the robotic system) may prime learners with a higher baseline of cognitive and procedural competence, which can then be applied when switching to manual techniques. Mastering complex tasks is typically more time-consuming and imposes a greater cognitive load, yet it can foster a deeper understanding of the underlying principles [18]. However, the interpretation of these results must account for the parallel progression in the fundamental task of performing a microvascular anastomosis itself, as well as the added complexity of switching from the conventional technique to the robotic system, which introduces an entirely new set of controls and lacks the familiar haptic feedback. Consequently, this pilot study does not allow a definitive conclusion regarding the optimal training sequence.

In both cohorts, we observed a rapid and consistent improvement in speed and microsurgical performance for both robot-assisted and hand-sewn anastomoses. Similar findings were reported in other preclinical studies evaluating the learning curve of RAMS [9,10,19], though those studies did not investigate the effect of the training sequence.

The RH group exhibited a more continuous and steeper learning curve with a significantly larger reduction in time per stitch and greater improvement in SAMS scores for robotic anastomoses in this group. We hypothesize that robotic assistance helps novices achieve accurate suture placement early on by compensating for underdeveloped fine motor skills, usually present in microsurgical beginners. This might enable the acquisition of declarative knowledge—such as optimal suture spacing and instrument positioning—before transitioning to manual techniques, where the participants primarily refine procedural execution.

This pattern aligns with motor learning frameworks distinguishing the initial cognitive (declarative) and later associative (procedural) phases of skill acquisition [20,21]. Robotic assistance might facilitate this shift by reducing execution demands and enabling focused conceptual learning. However, the proposed cognitive-transfer mechanism remains speculative. Alternative explanations include enhanced visuospatial adaptation or general task familiarization. Differences in learner confidence, engagement, or even subtle variations in instructor interaction could also contribute. Given the multifactorial nature of skill acquisition—spanning cognitive, motor, and perceptual domains—our findings, while suggestive, cannot definitively isolate causal mechanisms [22,23].

When examining the total time required to complete all ten anastomoses, we observed no statistically significant difference between the two cohorts. Upon assessing the final anastomoses in both groups, we found no significant difference in their quality, as measured by the SAMS score. However, the robot-assisted anastomoses still required significantly more time. Notably, the time difference decreased from 2.5-fold to 1.5-fold over the course of the study. However, anastomosis times did not reach a plateau, implying that additional improvements might have occurred with a greater number of procedures. Previous studies suggest that reaching such a plateau might take up to 16 anastomoses [3]. Our assessment, limited to five repetitions per technique, primarily captures the initial acquisition phase of learning a skill as complex as microsurgery, while still effectively highlighting initial adaptation patterns. Due to this short-term focus, it remains unclear whether the observed differences would persist, converge, or even reverse over longer training durations. In their preclinical study, Cho et al. found that arterial and venous anastomoses required significantly more time than conventional anastomosis, even after reaching a plateau and a comparable quality [10].

We observed a significantly larger reduction in anastomosis times in the RH group for both techniques, suggesting a steeper learning curve for that group. Von Reibnitz and colleagues evaluated the preclinical learning curve among microsurgeons and found a rapid increase in performance in an analysis of 117 anastomoses over three training sessions using the Symani surgical system [19]. They reported a significant improvement in anastomosis times and quality between the first and the third sessions. A correlation between microsurgical experience and anastomosis times, but not anastomosis quality, was found.

In a preclinical trial encompassing 180 anastomoses, Wessel et al. showed a steep learning curve among microsurgical novices, plastic surgery residents, and experts in microsurgery [9]. In their study, each participant performed ten anastomoses, alternating between RAMS and the hand-sewn technique. They found an association between RAMS usage and a lower frequency of errors as well as improved anastomosis quality. Interestingly, they reported significantly higher SAMS scores in the group of novices using RAMS than the conventional technique. Cho et al. demonstrated a steep preclinical learning curve among microsurgeons with fewer than five years of experience [10]. Comparable SARMS results between robot-assisted and conventional anastomoses were reached after four training sessions including eight anastomoses using rat femoral vessels.

In a previous clinical study, we found a shallow learning curve in the clinical application of RAMS for free flap transfer and peripheral nerve surgery [24,25]. The results of this study showed a steep learning curve in microsurgical novices in a synthetic model. We hypothesize that the slow rate of improvement in our clinical cases may be related to a plateau that was quickly reached either during the preclinical training or the clinical implementation phase. The steep preclinical learning curve in this study further supports this theory. Hence, it remains unclear whether further improvements in anastomotic times should be expected in the clinical setting.

We did not find statistically significant correlations between surgical experience proxies such as the participants’ previous experience with video games or their progression in medical school, in the German education system, which gradually exposes students to surgical fields. However, non-significant trends toward higher SAMS scores and lower times in advanced students suggest that prior surgical exposure may have an influence—though the small sample precludes firm conclusions.

Although great advances have been made in the field of supermicrosurgery, it still has not reached widespread acceptance [26]. In a literature review, Lo Torto et al. identified a total of only 1047 perforator-to-perforator free flap reconstructions reported in the literature up to 2022 [27]. Supermicrosurgical applications, such as lymphovenous and perforator-to-perforator anastomoses, have the potential to improve patient quality of life and reduce surgical morbidity, yet they are only slowly adopted into the mainstream of microsurgical practice. One reason for this slow adoption may be the high level of difficulty and the long training required to master the necessary skills. Robot-assisted microsurgery may serve as a catalyst for the widespread implementation of supermicrosurgery by potentially reducing the learning curve and skill requirements [10]. For this study, we employed a synthetic model with 2 mm vessels and 8-0 sutures to evaluate how the sequence of RAMS introduction affects learning. While this setup does not replicate the complexity of supermicrosurgery, it provides a controlled baseline from which to observe skill acquisition trends. The generalization of our findings to supermicrosurgical contexts, where RAMS may offer even greater benefits, remains uncertain. Bridging this gap will require several critical steps: validation of performance in supermicrosurgical models, comparative clinical studies assessing RAMS efficacy and outcomes in procedures such as lymphovenous or perforator-to-perforator anastomoses, and integration into structured training programs with defined competency benchmarks.

Additionally, while our findings were obtained using the Symani Surgical System, future studies should examine whether similar learning dynamics are observed across other robotic platforms like the MUSA or DaVinci systems. Given differences in instruments, control interfaces, and motion scaling, cross-platform generalizability needs to be critically assessed in future research.

Robotic assistance has been firmly established in other specialties such as gynecology and general surgery. The learning curve of robot-assisted laparoscopy has been extensively evaluated, where robotic assistance was found to be most advantageous [28]. When evaluating the clinical learning curve, a plateau in operating times was reached after 50 cases of robot-assisted laparoscopy for benign gynecological indications [29]. Heemskerk and colleagues evaluated the skill acquisition of surgical novices and found a flat learning curve and faster skill acquisition using the conventional technique [30]. However, it is important to note that, in contrast to microsurgery, robotic assistance in these specialties is employed for the entire operation rather than exclusively for the anastomosis.

Although our findings further enhance our understanding of the learning curve in RAMS and the microsurgical apprenticeship, the study nevertheless has certain limitations. The study was powered based on an expected SAMS score difference of 0.5 points (±0.3). However, the observed difference was smaller (0.4 points) and standard deviations were higher, reducing the actual power. As such, non-significant results may be due to insufficient power rather than the absence of an effect. A larger number of participants could have therefore improved the statistical power of our analyses. The SAMS score was slightly modified, and the absence of revalidation may have affected its applicability. The absence of assessor blinding is a key limitation, as it may have introduced detection, assessment, observer, or directional bias—potentially influencing scoring based on expectations about the robotic technique’s difficulty or efficacy. While observed improvements and cohort convergence suggest alignment with expected learning patterns, bias cannot be fully excluded. Future studies should implement blinded, multi-rater video assessments to enhance objectivity. Most participants did not reach a performance plateau, suggesting that additional improvement might have been observed had more than five attempts been completed. This limitation constrains our ability to draw conclusions about long-term skill retention or eventual performance equivalence between training sequences. As the study focused exclusively on microsurgical novices, the findings may not be generalizable to experienced microsurgeons. The standardized experimental setup does not account for key clinical factors—such as tissue handling, perfusion, and intraoperative bleeding—and may therefore not capture the full scope of the clinical learning curve. Future studies will therefore examine the preclinical learning curve of experienced microsurgeons.

## 5. Conclusions

Overall, our data demonstrate a steep preclinical learning curve for robot-assisted microsurgery in microsurgical novices. While participants who began with robotic assistance showed larger procedural gains, these findings are specific to a novice population using the Symani surgical system in a controlled, non-clinical setting. Robot-assisted microsurgery required more time per anastomosis, but we found no significant differences in microsurgical performance after ten anastomoses between the two techniques. Collectively, these results provide initial insight into training dynamics within this preclinical model; however, extrapolation to clinical practice should be undertaken with caution and confirmed in larger, future trials.

Future studies should not only examine the optimal sequencing of robotic and manual training but also consider extended protocols involving experienced surgeons and/or clinical settings. Based on our findings, we recommend incorporating blinded multi-rater assessments and exploring alternative outcome measures beyond SAMS—such as workload assessment using the NASA Task Load Index, anastomotic patency, or in vivo anastomosis with perfusion—to capture broader aspects of skill acquisition. Moreover, integrating objective performance metrics like quantitative motion analysis, for example, as proposed by Gholami et al. [31], could further enhance the robustness of future evaluations.

## Figures and Tables

**Table 1:** Baseline participant characteristics. Reported as n (%), unless otherwise stated.

Parameter	HR Group (n = 8)	RH Group (n = 8)
Age, Mean years ± SD	25.25 ± 1.91	23.63 ± 4.14
Year of medical school, median (IQR)	4 (3–5)	2.5 (1–4)
Male Sex	5 (62.5)	4 (50)
Ambidexterity	1 (12.5)	1 (12.5)
Right-handedness	7 (87.5)	7 (87.5)
Exposure to video games, median (IQR) *	2.5 (1.25–4)	2 (1.25–4.75)

* evaluated on a five-point rating scale (1 = none; 5 = very much).

**Table 2:** Anastomosis characteristics. Reported as mean ± SD unless otherwise stated.

Parameter	Hand-Sewn	Robotic	*p*-Value	Cohen’s d	95% CI
Time per stitch in min	4.1 ± 1.3	6.5 ± 2.6	**<0.0001**	**1.19**	**[1.5, 2.5]**
Total time in min	25.8 ± 8.3	40.5 ± 15.9	**<0.0001**	**1.15**	**[9.0, 16.0]**
No. of Stitches, median (IQR)	6 (6–7)	6 (6–7)	0.37	0.15	[0.0, 0.0]

**Table 3:** Time per stitch and SAMS score. Reported as mean ± SD unless otherwise stated. Purple background color stands for the anastomoses of the HR group, while a mint green background depicts the anastomoses of the RH group.

	Robotic (n = 8)	Manual (n = 8)	*p*-Value	Cohen’s d	95% CI
**Anastomosis 1**					
time per stitch in min	10.72 ± 2.04	5.83 ± 1.55	**0.002**	2.024	**[−7.57, −2.21]**
SAMS score	2.13 ± 0.64	2.88 ± 0.84	0.12	1.008	[0.0, 2.0]
**Anastomosis 5**					
time per stitch in min	*5.09 ± 1.35*	*3.47 ± 0.75*	**0.013**	1.485	**[−2.82, −0.42]**
SAMS score	3.75 ± 0.71	4.0 ± 0.54	0.50	0.40	[0.0, 1.0]
**Anastomosis 6**					
time per stitch in min	7.34 ± 3.31	4.24 ± 0.74	**0.0045**	1.293	**[1.1, 5.0]**
SAMS score	2.88 ± 0.84	3.75 ± 0.46	0.05	1.297	[−2.0, 0.0]
**Anastomosis 10**					
time per stitch in min	4.27 ± 0.9	2.92 ± 0.66	**0.0048**	1.706	**[0.49, 2.21]**
SAMS score	4.0 ± 0.54	4.38 ± 0.74	0.25	0.579	[−1.0, 0.0]

**Table 4:** Change in the average time per stitch, SAMS score, and number of errors between the first and last anastomosis. Reported as mean change (relative change in percent) unless otherwise stated. Purple background color stands for the anastomoses of the HR group, while a mint green background depicts the anastomoses of the RH group.

	HR Group (n = 8)	RH Group (n = 8)	*p*-Value	Cohen’s d	95% CI
**Robotic anastomoses**					
time per stitch in min	−3.1 (41.8)	−5.6 (52.6)	**0.033**	0.909	**[0.3, 5.9]**
SAMS score	1.1 (39.1)	1.6 (76.5)	0.22	0.858	[−1.0, 0.0]
number of errors	−1.2 (27.8)	−2.4 (48.7)	0.06	1.074	[0.0, 3.0]
**Manual anastomoses**					
time per stitch in min	−2.4 (40.56)	−1.3 (31.2)	**0.049**	1.077	**[−2.1, −0.01]**
SAMS score	1.1 (39.13)	0.6 (16.7)	0.37	0.633	[0.0, 1.0]
number of errors	−2 (50.0)	−0.875 (35.0)	0.22	0.643	[0.8, 3.0]

## Data Availability

The data presented in this study are available on request from the corresponding author.

## Abbreviations

The following abbreviations are used in this manuscript:

LVA	Lymphovenous anastomosis
RAMS	Robot-assisted microsurgery
SAMS	Structured Assessment of Microsurgical Skills
SARMS	Structured Assessment of Robotic Microsurgical Skills
